# Promotion of a neurosurgical academic journal on social media: a 1-year experience

**DOI:** 10.1007/s00701-023-05829-7

**Published:** 2023-10-16

**Authors:** Elena L. Sorba, Victor E. Staartjes, Carlo Serra, Luca Regli, Alex Alamri, Katrin Rabiei, Laura Lippa, Claire Karekezi, Angelos Kolias, Tiit Mathiesen

**Affiliations:** 1 Machine Intelligence in Clinical Neuroscience (MICN) Laboratory, Department of Neurosurgery, Clinical Neuroscience Center, University Hospital Zurich, University of Zurich, Zurich, Switzerland; 2 Department of Neurosurgery, The Royal London Hospital, Barts Health NHS Trust, London, UK; 3 Institution of Neuroscience & Physiology, Sahlgrenska Academy, Gothenberg, Sweden; 4 Dept. of Neurosurgery, Azienda Ospedaliero-Universitaria Senese, Siena, Italy; 5 Department of Neurosurgery, Rwanda Military Hospital, Kigali, Rwanda; 6 Division of Neurosurgery, Addenbrooke’s Hospital, University of Cambridge, Cambridge, UK; 7 University of Copenhagen, Copenhagen, Denmark; 8 Rigshospitalet, Copenhagen, Denmark; 9 Karolinska Intitutet, Stockholm, Sweden

**Keywords:** Social media, Twitter, Bibliometrics, Neurosurgery, Altmetrics

## Abstract

**Background:**

Social media (SoMe) use, in all of its forms, has seen massively increased throughout the past two decades, including academic publishing. Many journals have established a SoMe presence, yet the influence of promotion of scientific publications on their visibility and impact remains poorly studied. The European Journal of Neurosurgery «Acta Neurochirurgica» has established its SoMe presence in form of a Twitter account that regularly promotes its publications. We aim to analyze the impact of this initial SoMe campaign on various alternative metrics (altmetrics).

**Methods:**

A retrospective analysis of all articles published in the journal *Acta Neurochirurgica* between May 1st, 2018, and April 30th, 2020, was performed. These articles were divided into a historical control group — containing the articles published between May 1st, 2018, and April 30th, 2019, when the SoMe campaign was not yet established — and into an intervention group. Several altmetrics were analyzed, along with website visits and PDF downloads per month.

**Results:**

In total, 784 articles published during the study period, 128 (16.3%) were promoted via Twitter. During the promotion period, 29.7% of published articles were promoted. Overall, the published articles reached a mean of 31.3 ± 50.5 website visits and 17.5 ± 31.25 PDF downloads per month. Comparing the two study periods, no statistically significant differences in website visits (26.91 ± 32.87 vs. 34.90 ± 61.08, *p* = 0.189) and PDF downloads (17.52 ± 31.25 vs. 15.33 ± 16.07, *p* = 0.276) were detected. However, overall compared to non-promoted articles, promoted articles were visited (48.9 ± 95.0 vs. 29.0 ± 37.0, *p* = 0.005) and downloaded significantly more (25.7 ± 66.7 vs. 16.6 ± 18.0, *p* = 0.045) when compared to those who were not promoted during the promotion period.

**Conclusions:**

We report a 1-year initial experience with promotion of a general neurosurgical journal on Twitter. Our data suggest a clear benefit of promotion on article site visits and article downloads, although no single responsible element could be determined in terms of altmetrics. The impact of SoMe promotion on other metrics, including traditional bibliometrics such as citations and journal impact factor, remains to be determined.

## Introduction

The use of social media (SoMe) to promote published research has become popular in recent years [4–7, 9, 19, 21]. Various platforms such as LinkedIn, Instagram, Facebook, and Twitter are being used by scientific journals to share news and recent publications. SoMe has allowed journals to interact more closely with their readers by receiving direct feedback through likes, comments, and reposts. Twitter [11] has been the most discussed as a scientific SoMe platform, although there is still a limited amount of information about the influence of SoMe campaigns on article views, downloads, and citations [2].

*Acta Neurochirurgica* joined SoMe through Twitter (@ActaNeuro) in April 2019. A motivated board of SoMe editors was founded, and selected studies were posted in a standardized campaign. Within just 1 year, the journal’s Twitter account has put out over 400 tweets and, in December 2021, the account has increased from zero to over 3100 followers. The goal of this publication is to describe a 1-year experience in using SoMe to promote publications in our journal, and to study the effects of this SoMe campaign on the exposure of the published works.

## Materials and methods

### Design

A retrospective analysis of all articles published in the European Journal of Neurosurgery *Acta Neurochirurgica* between May 1st, 2018, and April 30th, 2020, was carried out. These articles were split into a historical control group — containing the articles published between May 1st, 2018, and April 30th, 2019, when the SoMe campaign was not yet established — and into an intervention group. The intervention group consisted of articles published between May 1st, 2019, and April 30th, 2020, representing articles published after the SoMe account had been established. April 2019 was a phase of adaptation to the new campaign. Therefore, the articles published during April 2019 were included into the historical control group. In addition, we analyzed the three published articles that reached the highest engagement rate (defined as engagement [clicks, likes, retweets, follows, and comments] divided by the total number of impressions).

### Criteria for inclusion

All papers published in *Acta Neurochirurgica* between 1 May 2018 and 30 April 2020 were included in this study.

### Intervention

The articles published on the twitter account were selected by the SoMe editorial team. There were no specific set criteria for the decision on whether an article was posted or not. The tweets included the title of the article, a link to the journal’s online publication, and if available an eye-catching figure or table from the article and standardized hashtags such as: #nsgy, #neurosurgery, #SoMe4Surgery, and #OnlineFirst. If possible, the authors’ or institutions’ twitter account were also tagged in the tweet.

### Outcome measures

The number of likes, retweets, comments, link clicks, and overall engagement, as well as impressions, and month of publication were captured for each article promoted on SoMe. For all papers published in *Acta Neurochirurgica* between May 1st, 2018, and April 30th, 2020, including the historical control and intervention groups, the number of full text PDF downloads and article website visits of each respective article at each month following online publication for the duration of the study period, were kindly provided by Springer Nature.

To account for the different follow-up lengths of articles published early vs. those published late within the study period, we divided the total number of website visits/PDF downloads and divided by the number of months for which the article was followed up with. For the few articles with more than one promotion, the post with the highest engagement overall was selected.

### Statistical analysis

Descriptive analysis was carried out on the Twitter statistics such as likes, retweets, and impressions. Continuous variables were given as means ± standard deviations (SD), and categorical variables as numbers (percentages). The exact version of Wilcoxon’s rank-sum test based on the “shift” algorithm described by Streitberg and Röhmel [27] and the Kruskal–Wallis test was used to compare continuous variables. Pearson’s Chi square test was applied for categorical variables. Trends in engagement statistics over quarters of the promotion period (3-month periods, starting from the initial date of SoMe promotion in May 2019) were statistically tested for in the Twitter subgroup in a two-tailed fashion. The Cochran-Armitage test was applied for dichotomous variables, and the exact version of the Jonckheere-Terpstra test, based on 10,000 permutations, was applied for continuous variables. Lastly, correlation among Twitter engagement statistics and website visits or full text PDF downloads was assessed using Spearman’s rank correlation. In accordance with Hinkle et al. [10], a correlation below 0.30 was interpreted as negligible. A *p* ≤ 0.05 was being regarded as statistically significant. R Version 4.1.1 was used for the analyses [24].

## Results

Table 1 provides an overview of all included articles, and Table 2 demonstrates the development of altmetrics over the course of the promotion period. From a total of 784 articles published during the study period, 128 (16.3%) were promoted via Twitter. During the promotion period, 29.7% of published articles were promoted. Overall, the published articles reached a mean of 31.3 ± 50.5 website visits and 17.5 ± 31.25 PDF downloads per month.

**Table 1:** Summary of article characteristics over both study periods

	Overall	Period 1 (no SoMe)	Period 2 (SoMe)	*P*
*Number of papers*	784	353	431	
Promoted papers (%)	128 (16.3)	0 (0)	128 (29.7)	-
Open access (%)	109 (13.9)	43 (12.2)	66 (15.3)	0.247
Article type (%)				0.643
Acknowledgments	2 (0.3)	1 (0.3)	1 (0.2)	
Book review	19 (2.4)	8 (2.3)	11 (2.6)	
Brief communication	21 (2.7)	10 (2.8)	11 (2.6)	
Editorial notes	47 (6.0)	26 (7.4)	21 (4.9)	
Erratum	14 (1.8)	8 (2.3)	6 (1.4)	
Letter	78 ( 9.9)	40 (11.3)	38 (8.8)	
Original paper	567 (72.3)	245 (69.4)	322 (74.7)	
Review paper	36 (4.6)	15 ( 4.2)	21 (4.9)	
Quarter of the year (%)				0.164
January–March	222 (28.3)	103 (29.2)	119 (27.6)	
April–June	197 (25.1)	81 (22.9)	116 (26.9)	
July–September	156 (19.9)	81 (22.9)	75 (17.4)	
October–December	209 (26.7)	88 (24.9)	121 (28.1)	
Website visits (mean (SD))	31.30 (50.50)	26.91 (32.87)	34.90 (61.08)	0.189
PDF downloads (mean (SD))	17.52 (31.25)	15.33 (16.07)	19.31 (39.49)	0.276

*SoMe* Social media, *SD* standard deviation

**Table 2:** Trends in engagement statistics of promoted articles over quarters of the promotion period

Quarter of the promotion period								
	Overall	1	2	3	4	Trend	Effect size	*P* (trend)
*Number of papers*	128	52	41	17	18			
Tags (%)	31 (24.2)	11 (21.2)	12 (29.3)	5 (29.4)	3 (16.7)	-	*Z* = 0.048	0.962
Media (%)	115 (89.8)	50 (96.2)	35 (85.4)	12 (70.6)	18 (100.0)	-	*Z* = 0.808	0.419
Likes (mean (SD))	12.48 (8.47)	12.10 (7.75)	13.63 (7.40)	10.65 (6.97)	12.72 (13.21)	-	JT = 2691	0.509
Retweets (mean (SD))	9.11 (5.83)	9.17 (6.49)	9.88 (4.45)	7.71 (4.41)	8.50 (7.66)	-	JT = 2578.5	0.240
Comments (mean (SD))	0.16 (0.50)	0.19 (0.66)	0.17 (0.38)	0.06 (0.24)	0.17 (0.38)	-	JT = 2869.5	0.869
Impressions (mean (SD))	3231.50 (2075.16)	3222.38 (2224.88)	3248.10 (1908.19)	2682.06 (1198.60)	3738.94 (2612.53)	-	JT = 2918	0.754
Engagement (mean (SD))	164.00 (196.76)	154.85 (215.69)	149.88 (142.10)	123.24 (105.51)	261.11 (281.58)	-	JT = 3112.5	0.253
Link clicks (mean (SD))	30.51 (35.55)	21.77 (18.10)	29.78 (39.87)	39.76 (41.58)	48.67 (49.21)	Increasing	JT = 3414	0.014*
Website visits (mean (SD))	48.86 (95.01)	39.21 (87.76)	39.02 (52.68)	42.27 (28.73)	105.35 (181.90)	Increasing	JT = 3820	< 0.001*
PDF downloads (mean (SD))	25.73 (66.70)	27.65 (87.67)	16.41 (12.33)	14.24 (5.58)	52.28 (93.20)	Increasing	JT = 3849	<0.001*

*SD*, Standard deviation; *JT*, Jonckheere-Terpstra effect size

*p ≤ 0.05

### Influence of SoMe promotion

Comparing the two study periods, no statistically significant differences in website visits (26.91 ± 32.87 vs. 34.90 ± 61.08, *p* = 0.189) and PDF downloads (17.52 ± 31.25 vs. 15.33 ± 16.07, *p* = 0.276) were detected. However, promoted articles had a significantly higher number of website visits (48.9 ± 95.0 vs. 29.0 ± 37.0, *p* = 0.005) and PDF downloads (25.7 ± 66.7 vs. 16.6 ± 18.0, *p* = 0.045) when compared to those not promoted during the promotion period (Table 3).

**Table 3:** Factors associated with website visits and PDF downloads

	Website visits		PDF downloads	
*Entire period 2 (SoMe, N = 431)*				
	Value (mean (SD))	*P*	Value (mean (SD))	*P*
Promoted (mean (SD))		0.005*		0.045*
Yes (*N*=128)	48.9 (95)		25.7 (66.7)	
No (*N*=303)	29 (37)		16.6 (18)	
Open access (mean (SD))		<0.001*		<0.001*
Yes (*N*=66)	92.4 (77.8)		38 (28.3)	
No (*N*=365)	24.5 (51.2)		15.9 (40.3)	
Article type (mean (SD))		<0.001*		<0.001*
Acknowledgments (*N*=1)	7.5 (NA)		7.8 (NA)	
Bookreview (*N*=18)	35 (22)		18.9 (13.8)	
Brief communication (*N*=11)	18.8 (22.7)		13.2(13.8)	
Editorial notes (*N*=21)	89.3 (148.7)		42.9 (87.7)	
Erratum (*N*=6)	29.6 (4.7)		12.4 (3.7)	
Letter (*N*=38)	27.9 (50.5)		16.5 (30.9)	
Original paper (*N*=322)	31.1 (49.2)		17.3 (36.1)	
Review paper (*N*=21)	62.4 (98.4)		37 (44.5)	
*Promoted articles only (N = 128)*				
Tags		0.108		0.145
Yes (*N*=31)	63.9 (95.1)		25.5 (36.8)	
No (*N*=97)	44.1 (95)		25.8 (73.9)	
Media		0.322		0.937
Yes (*N*=115)	48 (97.3)		26.6 (70.1)	
No (*N*=13)	56.3 (74.5)		18.1 (16.9)	
	Rho	*P*	Rho	*P*
Link clicks	*r* = 0.24	0.007*	*r* = 0.3	<0.001*
Likes	*r* = 0.01	0.877	*r* = 0.06	0.519
Retweets	*r* = −0.01	0.906	*r* = 0.03	0.715
Impressions	*r* = 0.06	0.500	*r* = 0.12	0.183
Comments	*r* = 0.16	0.064	*r* = 0.17	0.049*
Engagement	*r* = 0.06	0.506	*r* = 0.15	0.087

*SD* Standard deviation, *SoMe* social media

The influence of categorical factors (SoMe promotion, open access status, article type, tag inclusion, and media inclusion) on website visits and PDF downloads was assessed using Wilcoxon’s rank-sum test and the Kruskal–Wallis test. Conversely, the influence of continous factors (link clicks, likes, retweets, impressions, comments, engagement) was evaluated using Spearman’s rank correlation

### Trend analysis

**Fig. 1:**
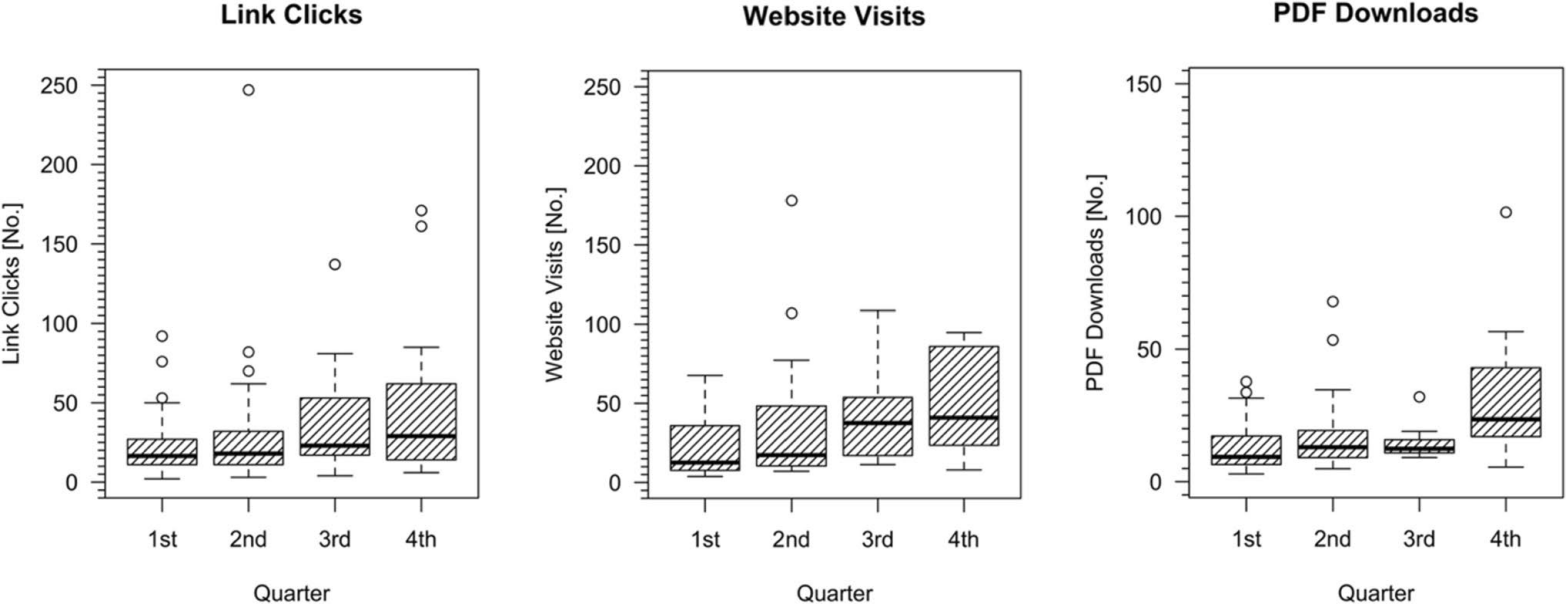
Boxplots illustrating the increase of link clicks, website visits, and PDF downloads over the quarters of promotion period. Extreme outliers have been removed

By evaluating Twitter engagement statistics and website visits as well as PDF downloads of highlighted articles, statistically significant trends could be shown for link clicks with an increase from 21.77 ± 18.1 to 48.67 ± 39.87 (*p* = 0.014), as well as website visits, which increased from 39.21 ± 87.76 to 105.35 ± 181.9 (*p* < 0.001) and PDF downloads, which increased from 16.41 ± 12.33 to 52.28 ± 93.2 (*p* < 0.001) over the 1-year promotion period (Fig. 1).

### Determinants of website visits and PDF downloads

Apart from the statistically significant influence of promotion on downloads and website visits, other factors correlating significantly with website visits and PDF downloads were article type (*p* <0.001) and open access status (*p* = 0.001). Looking solely at promoted articles, only link clicks showed a slight statistically significant correlation with PDF downloads (rho = 0.30) and a significant but negligible correlation with website visits (rho = 0.24). Authors tagged, number of comments, and attached media showed negligible influence on website visits or PDF downloads, as did engagement, likes, and retweets.

### Case Studies

**Fig. 2:**
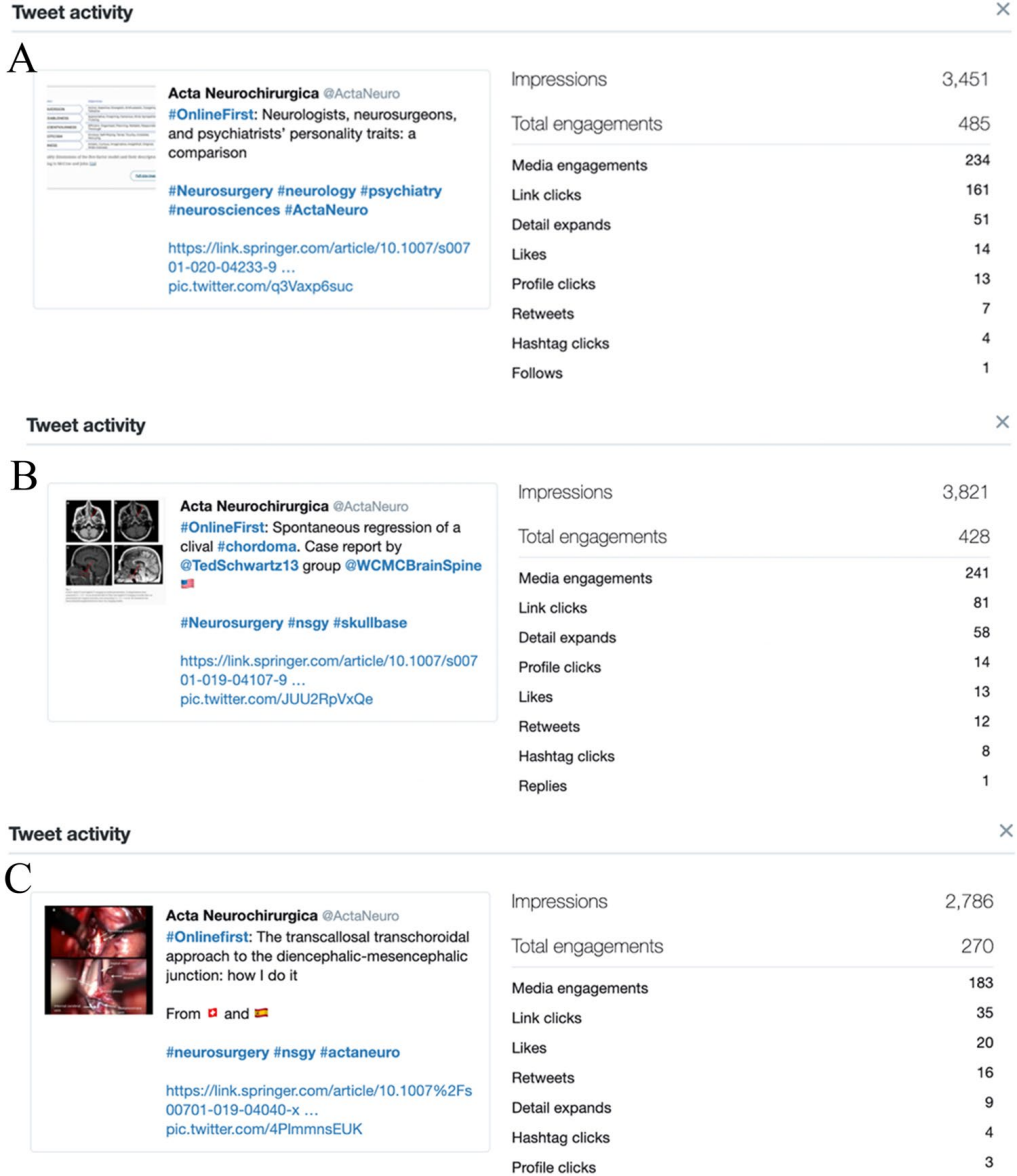
Examples of the three most successful tweets during the promotion period, selected according to engagement rate. Tweet A: Published on February 1st, 2020, with an engagement rate of 14.1%. Tweet B: Published on November 11th, 2019, with an engagement rate of 11.2%. Tweet C: Published on August 20th, 2019, with an engagement rate of 9.7%

The three tweets with the highest engagement rate were identified (Fig. 2). Tweet A about personality traits of neurologists, neurosurgeons, and psychiatrists, reached an engagement rate of 14.1%. Most engagements were either with media (indicating clicks to view the photo within the tweet) or link clicks to the article website, indicating a high interest in reading the complete article. Tweet B reached an engagement rate of 11.2% and deals with spontaneous regression of clival chordomas. In this case, most of the engagement was also with media, although the post received markedly more retweets than Tweet A — even though none of the tagged authors retweeted the post. Tweet C with an engagement rate of 9.7% is about a “How I do it” article explaining a microsurgical approach. Although this post registered markedly lower total engagement compared to the other two, it still has the highest number of likes and retweets out of the three.

## Discussion

During the first year of the Twitter campaign of the journal, as well as during the year before the start of the SoMe campaign, article website accesses, and fulltext downloads were analyzed. Although metrics were not significantly increased when comparing the promotion period overall with the pre-promotion period, the articles that were promoted registered twice the website visits and were more frequently downloaded — both statistically significantly increases when compared to articles that did not undergo SoMe promotion. This provides some indication that — while a social media campaign per se does not lead to overall greater engagement for the journal in its entirety — social media promotion of specific articles does lead to significantly increased reach of those specific articles.

Bibliometrics are defined as metrics such as citations or the impact factor of journals — both rather inert — have traditionally been used to measure the impact of scientific articles. More recently, article views and downloads have been used as a slightly more reactive metric. Newer impact measures, the so-called altmetrics [22] — alternative metrics, consisting of anything from SoMe activity, blog or media coverage, to activity on social bookmarking sites [3] — have been introduced as a tool to measure the influence and reach of an article throughout SoMe networks in near real-time [12, 28, 29]. In the future, we believe that altmetrics could increase in their importance as they will be a gauge that will show the scientific community their interest in an article alongside the citation rate. An increased understanding of the impact of SoMe campaigns by journals can consequently provide important guidance on which factors are most influential on both traditional bibliometrics and altmetrics. Hypothetically, if promotion on SoMe platforms by a journal were to lead to a significantly increased citation count or increased altmetrics, this would constitute a relevant bias and would also indicate that the visibility of articles is more strongly governed by SoMe promotion than by their intrinsic scientific value.

In contrast to our findings, earlier randomized controlled trials (RCT) did not show an increase in article views through SoMe promotion [4, 5]. However, newer studies show significant effects of SoMe promotion on full-text downloads and website visits [1, 17, 18, 30], although the effect on citations still remains to be determined. In a randomized study, Widmer et al. [30] found that promotion on Twitter, Facebook, and LinkedIn led to a significantly higher rate of accesses and download [30]. Similarly, Luc et al. [18] were able to show that tweeting does not only help with disseminating articles to a greater audience, but also significantly increases article citations within one year of publication [17, 18].

Nouri et al. [20] recently found that SoMe participation among European neurosurgery journals is still rather low compared to other specialties and compared to the North American community. We conclude from our findings, that the positive impact on the exposure of the published works is due to easier, direct access to the article site via Twitter ensured by the attached link, along with the expansion of the audience and faster dissemination through SoMe platforms as theorized before [7, 31]. It has also been shown that tweeting leads to a significant increase in article citations over time [15, 18, 31], whereas another study suggests the possibility of predicting the number of citations based on altmetrics [25]. Taken together, these points demonstrate how active SoMe promotion can be a valuable tool to invest in for both researchers and publishers.

Analyzing the entire promotion period, we were able to show that not only were our outcomes influenced by conventional factors such as article type and open access to the article, but also by the promotion itself. Especially the fact that open access status led to significantly greater reach is a testament to the importance of free access to knowledge and against paywalling. Consequently, the open access funding strategies that are being pursued by many governments could this appear to truly lead to a relevantly greater number of accesses. Though the impact of the promotion was not great enough to be seen when comparing the control period with the promotion period, the promoted articles still reached significantly more article site visits and article downloads.

To elicit which of the altmetrics was most relevant to exposure, we performed an in-depth analysis of the promoted articles and found solely a slight but mostly negligible but expected correlation between link clicks and article site visits as well as article downloads. Since none of the engagements by themselves could be shown to be the reason for the positive effect of promotion, we assume that the engagements as a whole contribute to the elevated exposure of the articles. This is nicely highlighted in our selected Tweets from the case study: each of them had provoked different reactions, be it by looking at the attached media or links, be it by retweeting, liking, or commenting on the post. Lastly, the impact of other, associated Twitter accounts who mention and retweet posts such as the publisher’s and the editorial office’s account are not to be underestimated.

No influence of attached media on our outcome measures was found, which agrees with a recently published study by Luc et al. [17], although they were able to show a trend towards increased link clicks in Tweets which incorporated media [17]. The influence of tags on a post was also not significantly correlated to either website visits or article downloads. Although the assumption that tagged author are more likely to promote their own publications may seem obvious, only few take advantage of this opportunity to promote their research — apart from the fact that most corresponding authors are not identifiable on Twitter or do not maintain a Twitter presence. A recent survey confirms our findings: Most of the surveyed authors were in support of SoMe promotion, although only few actually promoted their articles [8].

Investing in SoMe promotion may not only be beneficial to the reach of an article but may also influence traditional bibliometrics such as citations [18, 31]. The present landscape of scientific publishing, is characterized by volume growth [23] and proxy-parameters of scientific quality such as number of publications and journal’s impact-factors, that are increasingly questioned [14, 16]. In this context, SoMe appear to have assumed a role. The huge volume of available articles fosters new mechanisms to promote studies and gain attention. It is also becoming evident that scientific impact comes from other sources than citations and is not measurable just by impact-factors [26]. Further research into the impact of SoMe is warranted, including randomized analyses as well as studies incorporating other metrics and methodologies such as altmetrics or more rigorous statistics stemming from different or multiple sources such as CrossRef or PubMed, instead of the publisher solely. Similarly, a further increase in the significance of SoMe is to be expected, seen as the use of SoMe for promotion in the neurosurgical community, but also the medical and scientific community in general, still has potential for growth [20].

## Limitations

The strongest limitation of this study lies in its lack of randomization. Since the choice of article to promote was left up to the SoMe editors, their individual interests could have influenced the selection and biased results. Since the timepoint of posting has been shown to be of relevance to the reach of any post on SoMe [17], another limitation of the study lies in the editors ability to freely manage the account with no set time to post. This factor also led to periods of intense posting and other periods in which less content was uploaded, therefore again periodically limiting the reach of the posts and the journal’s rank in the Twitter algorithm that is strongly governed by activity. Lastly, interrupted time series analyses require special methodological considerations [13]: After careful consideration, the transitional period during initial establishment of the account was included in the historical control period, and we also tested for seasonality. Still, the design remains only quasi-experimental and controlled studies are necessary to elevate findings to a higher level of evidence.

## Conclusion

We report a 1-year initial experience with promotion of the European Journal of Neurosurgery *Acta Neurochirurgica* on Twitter. Although the impact of SoMe promotion on more traditional bibliometrics such as citations and journal impact factor remains to be determined, we have been able to show a clear benefit of promotion on article site visits and article downloads, although no single responsible element could be determined in terms of altmetrics. Our data provide some indication that — while a social media campaign per se does not lead to overall greater engagement for the journal in its entirety — social media promotion of specific articles does appear to lead to significantly increased reach of those promoted articles.

## Data Availability

The data in support of our findings can be obtained upon reasonable request from the corresponding author.
